# Comparative Analysis of Phytohormone Biosynthesis Genes Responses to Long-Term High Light in Tolerant and Sensitive Wheat Cultivars

**DOI:** 10.3390/plants13182628

**Published:** 2024-09-20

**Authors:** Zhi-Ang Li, Muhammad Fahad, Wan-Chang Li, Leeza Tariq, Miao-Miao Liu, Ya-Nan Liu, Tai-Xia Wang

**Affiliations:** 1 College of Life Sciences, Henan Normal University, Xinxiang 453007, China; li_zhi_ang2001@163.com (Z.-A.L.); miaomiao_liu@shbio.com (M.-M.L.); liuyanan@westlake.edu.cn (Y.-N.L.); wtaixia@sina.com (T.-X.W.); 2 Zhejiang Provincial Key Laboratory of Crop Genetic Resources, College of Agriculture and Biotechnology, Zhejiang University, Hangzhou 310058, China; muhammadfahad@zju.edu.cn; 3 Institute of Virology and Biotechnology, Zhejiang Academy of Agricultural Sciences, Hangzhou 310021, China; 4 National Key Laboratory for Rice Biology, Institute of Biotechnology, Zhejiang University, Hangzhou 310058, China; leeza_tariq@zju.edu.cn

**Keywords:** high light, phytohormones, gene expression, leaf senescence, *Triticum aestivum* L.

## Abstract

Phytohormones are vital for developmental processes, from organ initiation to senescence, and are key regulators of growth, development, and photosynthesis. In natural environments, plants often experience high light (HL) intensities coupled with elevated temperatures, which pose significant threats to agricultural production. However, the response of phytohormone-related genes to long-term HL exposure remains unclear. Here, we examined the expression levels of genes involved in the biosynthesis of ten phytohormones, including gibberellins, cytokinins, salicylic acid, jasmonic acid, abscisic acid, brassinosteroids, indole-3-acetic acid, strigolactones, nitric oxide, and ethylene, in two winter wheat cultivars, Xiaoyan 54 (XY54, HL tolerant) and Jing 411 (J411, HL sensitive), when transferred from low light to HL for 2–8 days. Under HL, most genes were markedly inhibited, while a few, such as *TaGA2ox*, *TaAAO3*, *TaLOG1*, and *TaPAL2*, were induced in both varieties. Interestingly, *TaGA2ox2* and *TaAAO3* expression positively correlated with sugar content but negatively with chlorophyll content and *TaAGP* expression. In addition, we observed that both varieties experienced a sharp decline in chlorophyll content and photosynthesis performance after prolonged HL exposure, with J411 showing significantly more sensitivity than XY54. Hierarchical clustering analysis classified the phytohormone genes into the following three groups: Group 1 included six genes highly expressed in J411; Group 2 contained 25 genes drastically suppressed by HL in both varieties; and Group 3 contained three genes highly expressed in XY54. Notably, abscisic acid (ABA), and jasmonic acid (JA) biosynthesis genes and their content were significantly higher, while gibberellins (GA) content was lower in XY54 than J411. Together, these results suggest that the differential expression and content of GA, ABA, and JA play crucial roles in the contrasting responses of tolerant and sensitive wheat cultivars to leaf senescence induced by long-term HL. This study enhances our understanding of the mechanisms underlying HL tolerance in wheat and can guide the development of more resilient wheat varieties.

## 1. Introduction 

Plants growing in natural environments frequently encounter light intensities that exceed their photosynthetic capacity [1]. This high light (HL) stress is often accompanied by heat stress (HS), creating a complex environmental challenge. In recent years, there has been a notable increase in extreme weather events combining HL and high-temperature conditions. This phenomenon is particularly evident during summer middays when temperatures can soar to between 30 °C and 40 °C, with light intensities reaching up to 2000 µmol m^−2^ s^−1^ [2]. Both intense light and elevated temperatures are significant abiotic stressors that can severely impact the photosynthetic pathway, consequently restricting plant growth and development. Under HL stress, photosynthetic reaction centers become saturated, and the surplus excitation energy can prove harmful, potentially causing irreversible damage to photosystem II (PSII) [3,4]. This damaging scenario results in photoinhibition, a persistent decrease in photosynthetic efficiency. Photoinhibition occurs due to an imbalance between the rate at which PSII is damaged and the rate at which it is repaired [5].

Plants exposed to excessive light energy beyond their photosynthetic capacity can experience photo-oxidative stress, particularly in emerging and senescing leaves [6]. In addition, phytohormones play critical roles in plant responses to HL stress. For instance, abscisic acid (ABA) has been shown to alleviate photoinhibition under various stresses and is crucial for HL stress tolerance in plants, as demonstrated by genetic studies in Arabidopsis and tomato that highlight the importance of ABA biosynthesis and signaling [7,8,9]. Furthermore, cytokinin (CK) is necessary for photoprotection against HL in Arabidopsis [10], while both CK and brassinosteroids (BR) enhance resistance to photo-inhibitory stress in tomato leaves [11]. Moreover, salicylic acid (SA) protects against HL and heat stress in wheat [12] and is involved in tomato responses to extreme HL [13]. In Arabidopsis, the interaction between gibberellic acid (GA) and ethylene plays a crucial role in apical hook development, with *DELLA* degradation leading to *ETHYLENE INSENSITIVE 3/EIN3-LIKE 1* (*EIN3/EIL1*) activation, which promotes *HOOKLESS1* (*HLS1*) expression and modulates hook formation in etiolated seedlings during light [14,15,16]. Likewise, jasmonic acid (JA) alleviated the negative effects of combined HL and HS by inducing several regulatory genes, viz., bZIP3, BHLH114, BHLH137, and WRKY8, which might allow plants to withstand heat and HL stress [17]. 

Leaf senescence, the final stage of leaf development, involves chlorophyll degradation and nutrient redistribution to younger plant parts and is accelerated by endogenous factors like leaf age as well as by environmental stresses [18]. Plant hormones play a crucial role in regulating leaf senescence, either promoting or repressing the process. Promoters include ABA, BR, ET, JA, and SA, while auxin, CK, and GA act as repressors. These hormones work in conjunction with complex networks of transcription factors, including bZIP, NAC, and WRKY families. Therefore, ET, a senescence-promoting hormone, binds to and inhibits the ETRs–CTR1 receptor–kinase complex, leading to *ETHYLENE INSENSITIVE 2* (*EIN2*) activation. Activated *EIN2* then stabilizes *EIN3* and related EIL transcription factors [19]. Abscisic acid (ABA) is another plant hormone that promotes leaf senescence. The core ABA signaling pathway involves the following three main components: *SnRK2s*, which activate group A bZIP transcription factors including *ABI5*; group A *PP2Cs*, which inhibit *SnRK2s*; and *PYR/PYLs*, which bind to ABA. When ABA binds to *PYR/PYLs*, it promotes their binding to *PP2Cs*, thus inhibiting *PP2C*, thus activating *ORE1* and the genes involved in chlorophyll degradation [20,21]. BR positively regulates senescence in Arabidopsis and *Carica papaya* but negatively regulates senescence in wheat [22,23,24]. Furthermore, different phytohormones may regulate specific types of senescence. SA may regulate developmental senescence, JA may regulate dark-induced senescence, and ET may regulate cell-suspension senescence [25]. This suggests that the roles of phytohormones in leaf senescence are context-dependent and influenced by multiple factors.

In field conditions, persistent photoinhibition or photo-oxidative stress induced by HL, especially when combined with other stressors like extreme temperature or drought, can accelerate leaf senescence in wheat during grain filling [26]. Long-term HL exposure (6–8 days) significantly reduces chlorophyll content, photochemical efficiency, and enzyme activities, while increasing various metabolites and oxidative stress markers [27]. The expression of wheat SAGs and accumulation of sugars increase with prolonged HL exposure, suggesting a link between sugar accumulation and HL-induced leaf senescence [27,28]. While it is known that phytohormone signaling pathways interact with sugars to regulate plant growth and development [16,29], the specific mechanisms of how these interactions regulate HL-induced leaf senescence remain unclear. Notably, HL tolerance in wheat is genotype-dependent and controlled by quantitative trait loci (QTL) and environmental factors [26,28,30,31,32]. For example, the winter wheat variety Xiaoyan 54 (XY54) exhibits better HL tolerance and slower senescence under long-term HL exposure compared to the more sensitive variety, Jing 411 (J411) [27,28]. 

Despite these insights, the roles of phytohormones in wheat responses to HL stress remain poorly understood, highlighting a need for further research in this important cereal crop. Therefore, the objective of this study was to explore the expressional responses of phytohormone biosynthesis genes to long-term HL in wheat and elucidate the possible roles of these genes in regulating HL stress tolerance in XY54. This research aims to contribute to our understanding of the complex interplay between phytohormones, HL stress, and leaf senescence in wheat, potentially informing future strategies for improving crop resilience to environmental stresses.

## 2. Results

### 2.1. Photosynthetic Pigment Content under Long-Term HL Stress

**Figure 1:**
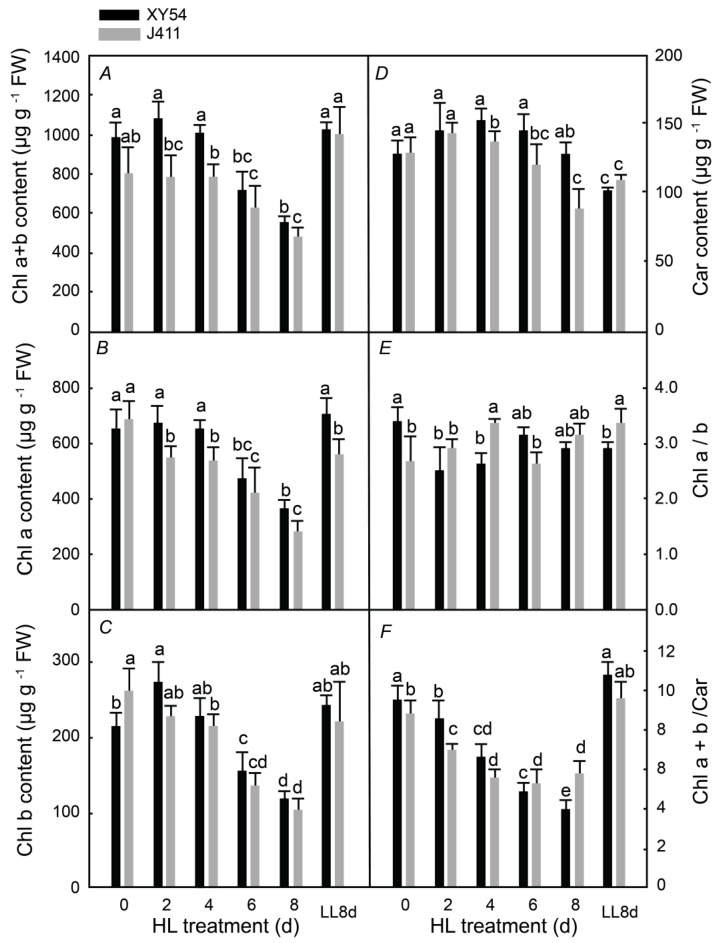
(**A**) total chlorophyll (Chl a + b); (**B**) chlorophyll a (Chl a); (**C**) chlorophyll b (Chl b); (**D**) carotenoid (Car) contents of wheat under sustained strong light stress; (**E**) changes in the ratio of chlorophyll a and chlorophyll b (Chl a/b); and (**F**) the ratio of total chlorophyll to carotenoid content (Chl a + b/Car). Data are expressed as mean ± standard deviation, and different letters indicate a significant level of *p* < 0.05.

To evaluate the photosynthetic pigment contents between XY54 and J411, the changes in chlorophyll and carotenoid content were measured after they were transferred from low light to HL treatment for 2–8 days (Figure 1). In the HL-grown plants, the total chlorophyll (Chl a + b), chlorophyll a, and chlorophyll b contents of both XY54 and J411 varieties decreased sharply on the 6th day of the HL treatment and dropped to the lowest levels by the 8th day. Conversely, the carotenoid content increased significantly on the 2nd day, remained high, but then dropped sharply by day 8, as shown in Figure 1A–D. The ratio of Chl a/b did not change significantly, but the ratio of Chl a + b/Car decreased sharply from the 2nd day in both varieties (Figure 1E,F). The seedlings were initially grown under LL conditions for 8 days, which was referred to as low light conditions for 8 days (LL8d) and served as the LL control. Compared to the LL8d control, during the 6th to 8th day of the HL treatment, the chlorophyll content decreased rapidly, leading to leaf senescence. Moreover, the chlorophyll content of J411 began to decrease on the 4th day of the HL treatment, two days earlier than the decrease observed in XY54. The interaction between the wheat variety and HL duration was significant, as evidenced by the earlier decline in chlorophyll content in J411 compared to XY54, particularly from day 4 to day 8 (*p* < 0.05). This suggests that the effect of HL stress on chlorophyll degradation is more severe in J411, indicating a higher sensitivity to HL-induced leaf senescence.

### 2.2. Assessment of Photosynthesis Performance under Long-Term HL Stress

**Figure 2:**
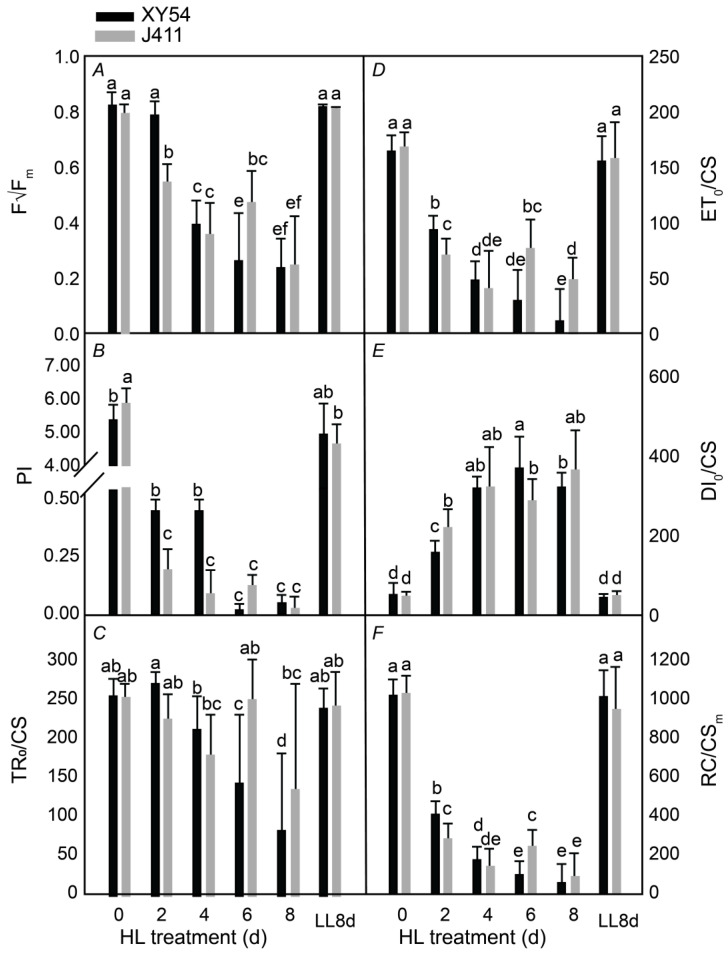
(**A**) maximum light quantum yield (F_v_/F_m_); (**B**) performance index (PI); (**C**) energy captured per unit reaction center (TR_o_/CS) of photosystem II under continuous strong light stress; (**D**) the energy transferred by electrons per unit reaction center (ET_o_/CS); (**E**) the energy dissipated per unit reflection center (DI_o_/CS); and (**F**) the number of open reaction centers per unit area (RC/CS_m_) response to bright light. Data are expressed as mean ± standard deviation, and different letters indicate a significant level of *p* < 0.05.

Next, we investigated the changes in the photochemical activity of both varieties under HL stress. Chlorophyll fluorescence parameters including Fv/Fm, PI, TRo/CS, ETo/CS, DIo/CS, and RC/CSm were measured in XY54 and J411. On the second day of HL treatment, both varieties exhibited a sharp decline in Fv/Fm (Figure 2A), PI (Figure 2B), ETo/CS (Figure 2D), and RC/CSm (Figure 2F), indicating significant inhibition of PSII photochemical activity and the onset of photooxidative stress. As the duration of HL treatment increased, Fv/Fm, PI, ETo/CS, and RC/CSm continued to decrease, reflecting further damage to the PSII system under prolonged HL stress. Additionally, TRo/CS in both varieties decreased significantly after 6–8 days of HL treatment (Figure 2C), suggesting that HL stress also inhibits energy capture in the PSII reaction center, which aligns with the observed trend of chlorophyll degradation. Concurrently, DIo/CS increased significantly from the second day of HL treatment, reaching its maximum value on the eighth day, demonstrating that continuous high light stress enhanced heat dissipation (Figure 2E). Consequently, the energy obtained by the PSII photochemical reaction center decreased, leading to a reduction in Fv/Fm and inhibition of photosynthesis. In addition, a significant interaction between variety and HL duration was observed for chlorophyll fluorescence parameters (*p* < 0.05). These parameters showed a more pronounced decline in J411, which reflects a greater inhibition of PSII under prolonged HL stress, suggesting that J411 suffered more photochemical damage compared to XY54 under the same conditions.

### 2.3. Effects of Long-Term HL Stress on GA Biosynthesis Pathway Genes and GA Content

**Figure 3:**
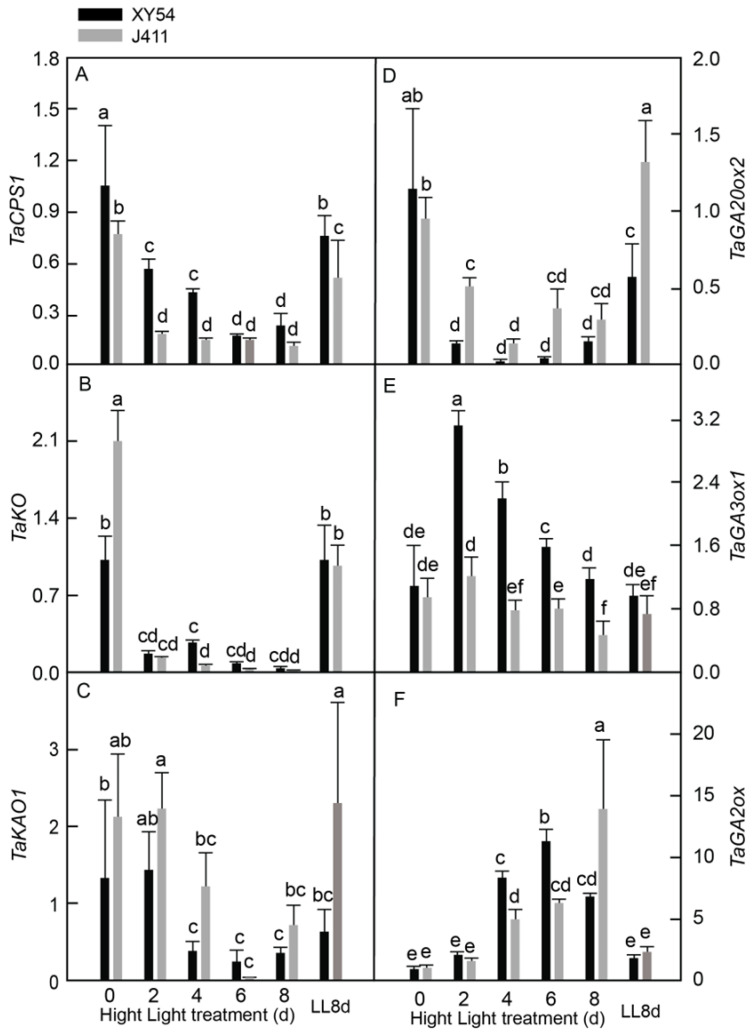
Expression responses of six GA biosynthesis-related genes encoding: (**A**) *ent-copalyl diphosphate synthase 1* (*TaCPS1*); (**B**) *ent-kaurene oxidase (TaKO)*; (**C**) *ent-kaurenoic acid oxidase 1 (TaKAO1)*; (**D**) *GA20-oxidase 2 (TaGA20ox2)*; (**E**) *GA3-oxidase 1 (TaGA3ox1)*; and (**F**) *GA2-oxidase (TaGA2ox)* in XY54 and J411 to leaf senescence induced by HL. The data are represented as mean ± SE. The different letters indicate a significant difference at *p* < 0.05.

To investigate the expressional responses of phytohormone biosynthesis genes GA content to long-term HL in wheat, we conducted a comprehensive study using plants of two varieties, XY54 and J411. These seedlings were transferred from LL to HL conditions for 2–8 days. As controls, seedlings grown under both HL0d and LL8d conditions were used. Our study focused on the following five key genes involved in GA metabolism: *ent-copalyl diphosphate synthase 1* (*TaCPS1*); *ent-kaurene oxidase* (*TaKO*); *ent-kaurenoic acid oxidase 1* (*TaKAO1*); *gibberellin 20 oxidase 2* (*TaGA20ox2*); *GA3-oxidase 1* (*TaGA3ox1*); and *GA2-oxidase* (*TaGA2ox*) (Figure 3). We observed that the expression levels of *TaCPS1* (Figure 3A), *TaKO* (Figure 3B), and *TaGA20ox2* (Figure 3D) declined significantly and consistently in both XY54 and J411 varieties throughout the 2–8 days DAT period. Similarly, *TaKAO1* expression in both varieties decreased substantially at 4–8 DAT (Figure 3C). Interestingly, *TaGA3ox1* exhibited a different pattern between the two varieties. In XY54, its expression increased at 2–6 DAT, followed by a decline to approximately LL control levels at 8 DAT. In contrast, J411 showed only slight changes in *TaGA3ox1* expression throughout the 2–8 DAT period (Figure 3E). For both varieties, a significant increase in *TaGA2ox* expression at 4–8 DAT was noted (Figure 3F). When comparing the two varieties, XY54 exhibited significantly higher expression levels of *TaCPS1* at 2–4 DAT and *TaGA3ox1* at 2–8 DAT. Conversely, XY54 showed lower expression levels of *TaKAO1* at 4 DAT and *TaGA20ox2* at 2 DAT and 6 DAT compared to J411. The expression levels of key GA biosynthesis genes, particularly *TaGA3ox1* and *TaGA2ox*, showed significant interaction effects between the variety and stress duration (*p* < 0.05). In XY54, *TaGA3ox1* expression increased between 2 and 6 days under HL, while J411 exhibited minimal change, suggesting that GA biosynthesis regulation differed between the two varieties in response to prolonged stress. In addition, GA accumulation was significantly lower in both XY54 and J411, suggesting valuable insights into the differential responses of GA biosynthesis genes and accumulation to HL stress in wheat varieties (Figure S1A).

### 2.4. JA Biosynthesis Gene Expression and Accumulation under Long-Term HL Stress

**Figure 4:**
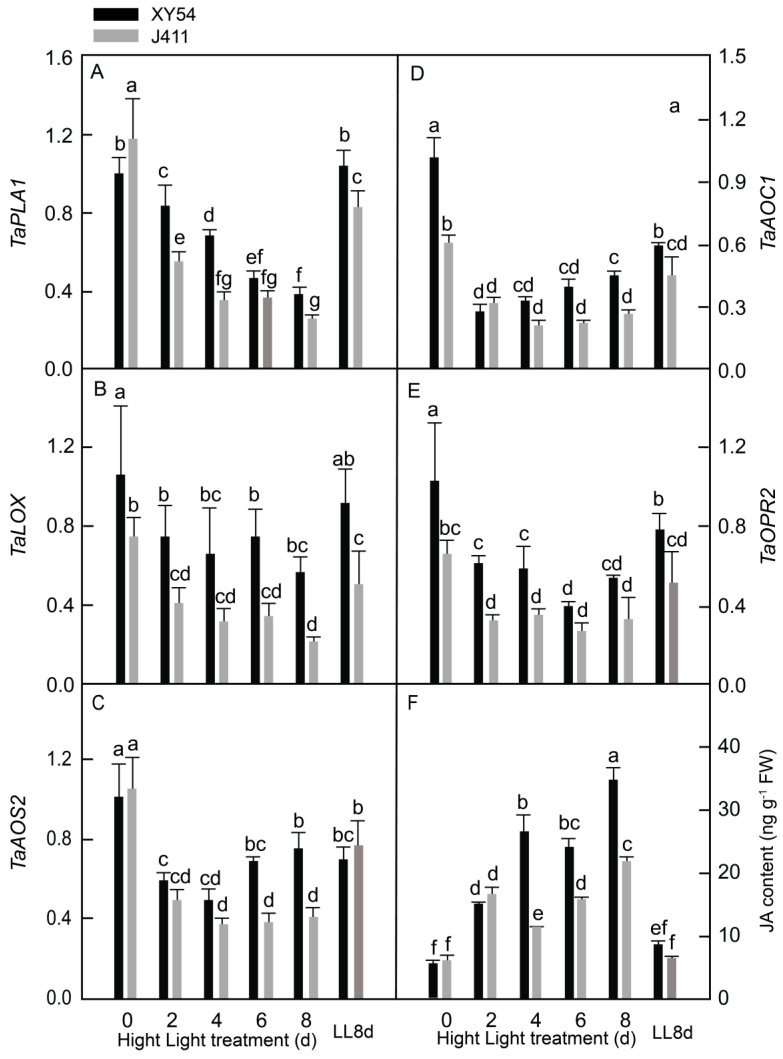
Eexpression responses of five JA biosynthesis-related genes encoding: (**A**) phospholipase A1 (*TaPLA1*); (**B**) lipoxygenase (*TaLOX*); (**C**) allene oxide synthase 2 (*TaAOS2*); (**D**) allene oxide cyclase 1 (*TaAOC1*); and (**E**) 12-oxophytodienoate 2 (*TaOPR2*); and as well as (**F**) JA content in XY54 and J411 responding to leaf senescence induced by HL. The data are represented as mean ± SE. The different letters indicate a significant difference at *p* < 0.05.

To further elucidate the intricate responses of phytohormones to HL stress in wheat, our attention was next focused on JA biosynthesis and accumulation. In this phase of the study, the expression patterns of five key JA biosynthesis genes were examined and JA content was measured in XY54 and J411 wheat varieties exposed to HL conditions (Figure 4). The genes analyzed included *phospholipase A1* (*TaPLA1*), *lipoxygenase* (*TaLOX*), *allene oxide synthase 2* (*TaAOS2*), *allene oxide cyclase 1* (*TaAOC1*), and *12-oxophytodienoate 2* (*TaOPR2*). These results revealed that, in both varieties, *TaPLA1* and *TaLOX* transcripts decreased significantly under HL compared to the LL controls. Furthermore, *TaAOS2* expression in J411 declined at 2–8 DAT, while in XY54, it decreased at 2–4 DAT compared to the LL8d control (Figure 4C). The expression of *TaAOC1* in XY54 was significantly lower at 2–8 DAT, whereas in J411, it remained approximate to the LL8d control (Figure 4D). Similarly, *TaOPR2* expression in J411 at 2–8 DAT did not significantly differ from the LL8d control, while in XY54, it was significantly lower at 6–8 DAT (Figure 4E).

### 2.5. Expression and Accumulation of ABA Biosynthesis Pathway Genes under Long-Term HL Stress

**Figure 5:**
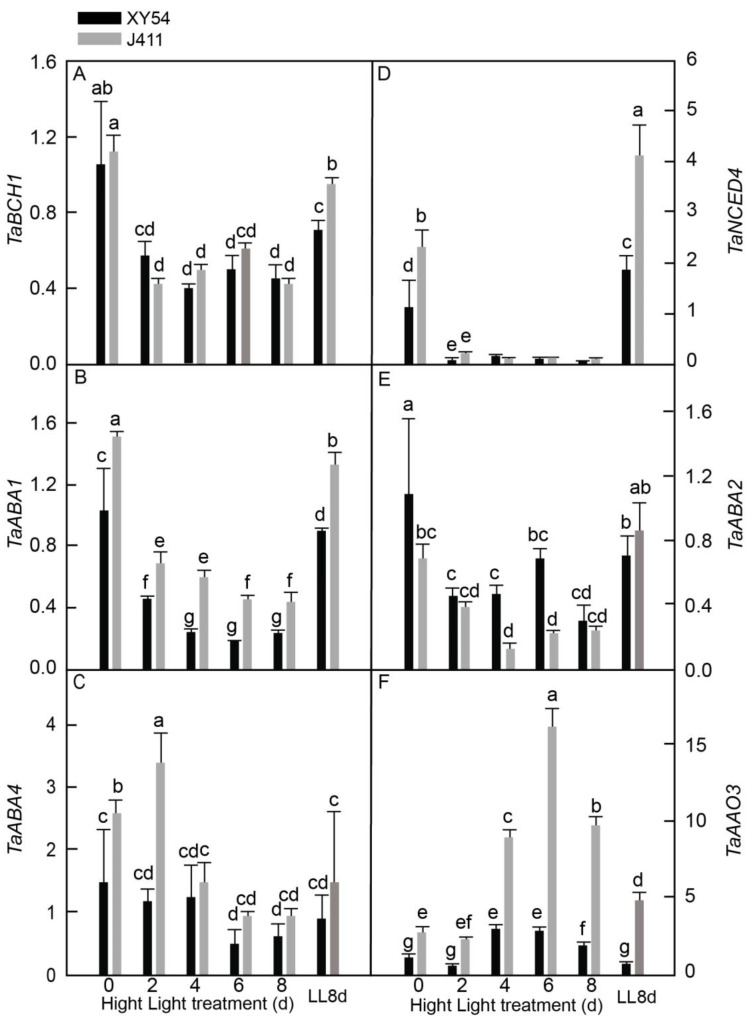
Expressional responses of six ABA biosynthesis-related genes encoding: (**A**) *β-carotene hydroxylase (TaBCH1)*; (**B**) *zeaxanthin epoxidase (TaABA1)*; (**C**) *ABA DEFICIENT 4 (TaABA4)*; (**D**) *9-cis-epoxy carotenoid dioxygenase (TaNCED1)*; (**E**) *short-chain alcohol dehydrogenase (TaABA2)*; and (**F**) *abscisic aldehyde oxidase (TaAAO3)* in XY54 and J411 to leaf senescence induced by HL. The data are represented as mean ± SE. The different letters indicate a significant difference at *p* < 0.05.

In the investigation of ABA biosynthesis gene responses and accumulation to HL stress, the mRNA transcripts of five key genes were analyzed in XY54 and J411 wheat varieties exposed to HL for 2–8 days. These genes included *β-carotene hydroxylase* (*TaBCH1*), *zeaxanthin epoxidase* (*TaABA1*, also known as *TaZEP*), *ABA DEFICIENT 4* (*TaABA4*), *9-cis-epoxy carotenoid dioxygenase* (*TaNCED1*), *short-chain alcohol dehydrogenase* (*TaABA2*), and *abscisic aldehyde oxidase* (*TaAAO3*) (Figure 5). Our results suggested that the expression of *TaBCH* (Figure 5A), *TaABA1* (Figure 5B), *TaNCED4* (Figure 5D), and *TaABA2* (Figure 5E) in both varieties consistently declined at 2–8 DAT, with *TaNCED* transcripts appearing to be the most sensitive to HL conditions. Interestingly, *TaABA4* expression in J411 increased significantly at 2 DAT, followed by a decrease to the LL8d control level at 4–8 DAT, while in XY411, it changed only slightly compared to the LL8d control (Figure 5C). In contrast, *TaAAO3* expression levels increased substantially at 4–6 DAT in both varieties (Figure 5F). When comparing the two varieties, it was observed that XY54 exhibited significantly higher expression of *TaABA2* at 4–6 DAT but lower expression of *TaABA1* at 2–8 DAT and lower expression of *TaABA4* at 2 DAT at all sampling time points relative to J411. Notably, no significant difference was observed in *TaABA4* expression at 8 DAT and LL8d for both varieties, suggesting that this gene expression was regulated by leaf age rather than HL stress. Additionally, ABA content was increased in both varieties under HL stress conditions, as it helps to regulate plant responses to stress by promoting HL tolerance (Figure S1B).

However, significant interactions were noted in the expression of JA and ABA biosynthesis genes, particularly *TaAOS2* and *TaAAO3* (*p* < 0.05), where the expression patterns varied between varieties as HL stress increased. This interaction is crucial because it highlights how JA and ABA contribute to stress tolerance differently in each variety.

### 2.6. Differential Expressional Responses of CK and ET Biosynthesis Pathway Genes under Long-Term HL Stress

**Figure 6:**
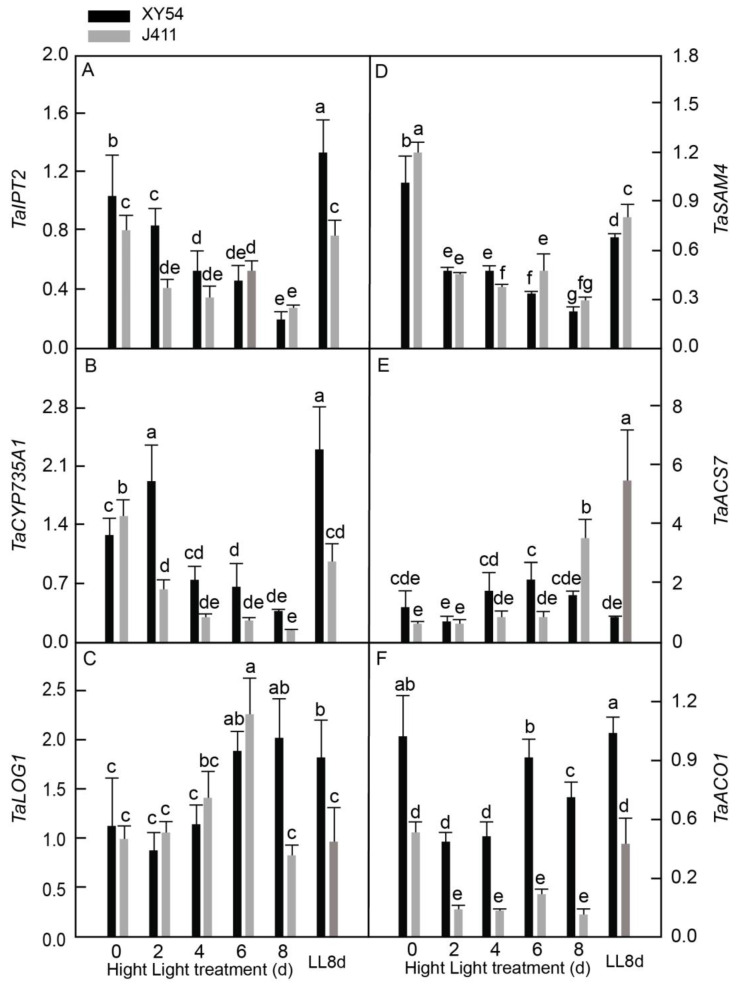
Expressional responses of CK and ET biosynthesis-related genes encoding: (**A**) *tRNA isopentenyl transferase 2 (TaIPT2)*; (**B**) *cytokinin hydroxylase (TaCYP735A1)*; (**C**) *cytokinin riboside 5′-monophosphate phosphoribohydrolase 1 (TaLOG1)*; (**D**) *S-adenosylmethionine synthetase 4 (TaSAM4)*; (**E**) *1-aminocyclopropane-1-carboxylic acid synthase 7 (TaACS7)*; and (**F**) *1-aminocyclopropane-1-carboxylic acid oxidase 1 (TaACO1)* in XY54 and J411 to leaf senescence induced by HL. The data are represented as mean ± SE. The different letters indicate a significant difference at *p* < 0.05.

Similarly, the expression patterns of key CK and ET biosynthesis genes were measured in XY54 and J411 wheat varieties exposed to HL conditions (Figure 6). For CK biosynthesis, the focus was on *tRNA isopentenyltransferase* (*TaIPT2*), *cytokinin hydroxylase* (*TaCYP735A1*), and *cytokinin riboside 5′-monophosphate phosphoribohydrolase 1* (*TaLOG1*). Together with this, ET biosynthesis genes, including *S-adenosylmethionine synthetase 4* (*TaSAM4*), *1-aminocyclopropane-1-carboxylic acid synthase 7* (*TaACS7*), and *1-aminocyclopropane-1-carboxylic acid oxidase 1 (TaACO1),* were investigated. It was shown that *TaIPT2* expression levels declined significantly from 2 DAT in both varieties, reaching their lowest values at 8 DAT (Figure 6A). Interestingly, *TaCYP735A1* expression exhibited contrasting patterns between the two varieties. In J411, it decreased drastically at 2–8 DAT, while in XY54, it initially increased and peaked at 2 DAT before drastically decreasing at 6–8 DAT (Figure 6B). *TaLOG1* expression in XY54 was elevated significantly at 6–8 DAT, whereas in J411, it increased at 6 DAT before returning to LL control levels (Figure 6C). Notably, we found no significant difference in *TaLOG1* expression between 8 DAT and LL8d in either variety, suggesting potential regulation by leaf age rather than HL stress alone. In the comparative examination, XY54 exhibited significantly higher expression levels of *TaIPT2* at 2 DAT, *TaCYP735A1* at 2 DAT, and *TaLOG2* at 8 DAT compared to J411. Regarding ET biosynthesis genes, *TaSAM4* expression in both XY54 and J411 declined markedly at 2–8 DAT (Figure 6D). *TaACS7* expression in J411 increased significantly at 8 DAT compared to 0 DAT, although it remained significantly lower than the LL8d control; however, in XY54, it changed only slightly (Figure 6E). Similar to *TaSAM4*, *TaAOC1* expression in both varieties declined at 2–8 DAT, except for an increase to approximately LL control levels at 6 DAT in XY54 (Figure 6F). Furthermore, XY54 had a significantly higher expression of *TaACS7* at 6 DAT and *TaAOC1* at 2–8 DAT, but a lower expression of *TaSAM4* and *TaACS7* at 6 DAT and 8 DAT, respectively, when compared to J411.

### 2.7. Expressional Dynamics of SA and BR Biosynthesis Pathway Genes under Long-Term HL Stress

**Figure 7:**
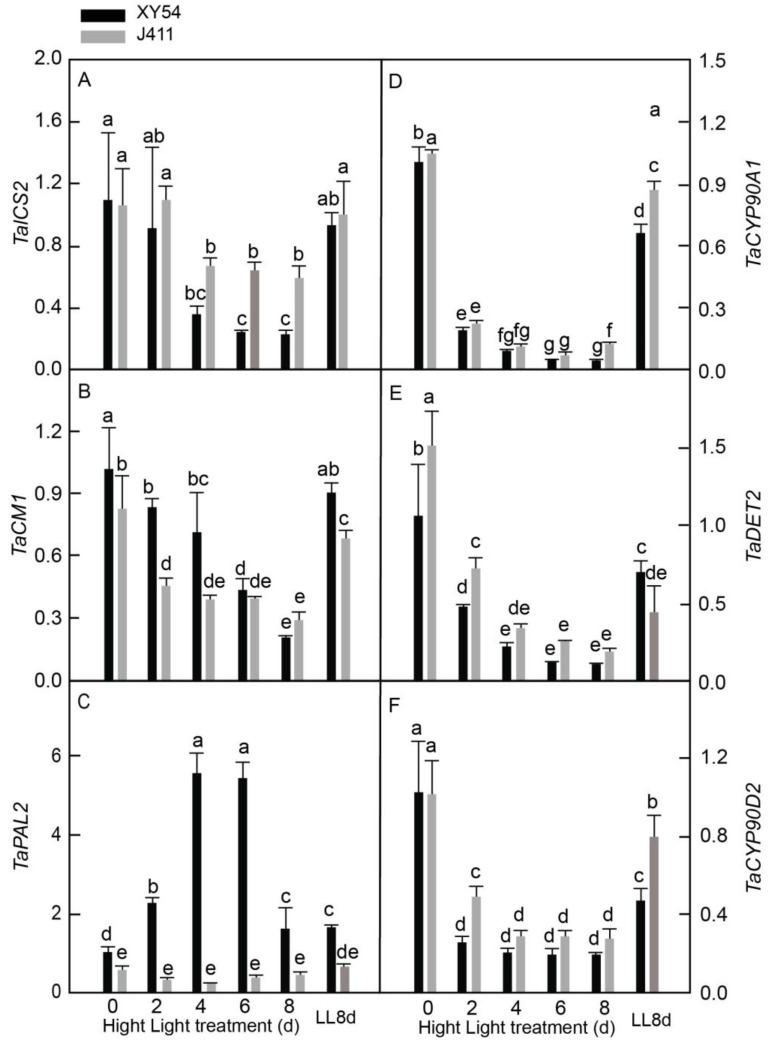
Expressional responses of SA and BR biosynthesis-related genes encoding: (**A**) *isochorismate synthase (TaICS2)*; (**B**) *chorismate mutase (TaCM1)*; (**C**) *phenylalanine ammonia-lyase (TaPAL1);* (**D**) *cytochrome P450 90A1 (TaCYP90A1)*; (**E**) *steroid 5-α-reductase (TaDET2)*; and (**F**) *cytochrome P450 90D2 (TaCYP90D2)* in XY54 and J411 to leaf senescence induced by HL. The data are represented as mean ± SE. The different letters indicate a significant difference at *p* < 0.05.

To investigate the role of SA and BR, the transcripts of three SA biosynthesis genes—*isochorismate synthase* (*TaICS2*), *chorismate mutase* (*TaCM1*), and *phenylalanine ammonia-lyase* (*TaPAL1*)—and three BR biosynthesis genes—*cytochrome P450 90A1* (*TaCYP90A1*), *steroid 5-α-reductase* (*TaDET2*), and *cytochrome P450 90D2* (*TaCYP90D2*) were analyzed in XY54 and J411 wheat varieties subjected to HL conditions (Figure 7). The results revealed that the expression of *TaICS2* (Figure 7A) and *TaCM1* (Figure 7B) in both varieties declined significantly at 4–8 DAT and 2–8 DAT, respectively, while *TaPAL2* expression in XY54 increased and peaked at 4–6 DAT, followed by a decrease to the LL control level, whereas it changed very slightly in J411 (Figure 7C). Notably, no significant difference in *TaPAL2* expression between 8 DAT and the LL8d control was observed for both varieties, suggesting that leaf age may play a role in regulating *TaPAL2* expression. In our comparative analysis, we found that XY54 exhibited higher expression of *TaCM1* at 2–4 DAT and *TaPAL2* at all sampling time points but lower expression of *TaICS2* at 4–8 DAT, as well as lower expression of *TaDET2* and *TaCYP90D2* at 2 DAT compared to J411. Regarding the expression of the three BR biosynthesis genes (*TaCYP90A1*, *TaDET2*, and *TaCYP90D2*), all decreased considerably in both XY54 and J411 at 2–8 DAT, with almost no significant differences between the two varieties under HL conditions (Figure 7D–F).

### 2.8. Expressional Responses of Auxin, Nitric Oxide, and Strigolactone Biosynthesis Pathway Genes under Long-Term HL Stress

**Figure 8:**
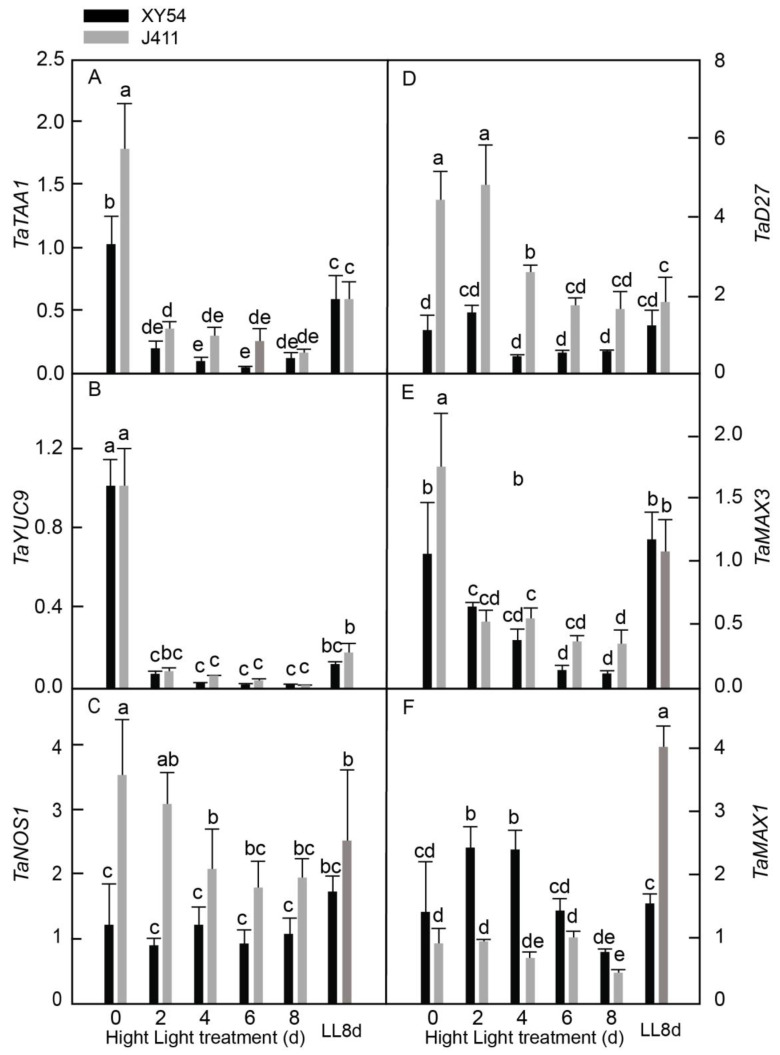
Expressional responses of indole-3-acetic acid, nitric oxide, and strigolactone biosynthesis-related genes encoding: (**A**) *L-tryptophan--pyruvate aminotransferase 1* (*TAA1*); (**B**) *indole-3-pyruvate monooxygenase YUCCA9 (TaYUC9*); (**C**) *nitric oxide synthase 1* (*TaNOS1*); (**D**) *β-carotene isomerase D27* (*TaD27*); (**E**) *carotenoid cleavage dioxygenase 7* (*TaMAX3*); and (**F**) *cytochrome P450 711A1* (*TaMAX1*) in XY54 and J411 to leaf senescence induced by HL. The data are represented as mean ± SE. The different letters indicate a significant difference at *p* < 0.05.

Next, the biosynthesis pathways of IAA, NO, and SL was investigated in XY54 and J411 wheat varieties’ responses to HL stress (Figure 8). For IAA biosynthesis, we focused on *L-tryptophan--pyruvate aminotransferase* (*TaTAA1*) and *indole-3-pyruvate monooxygenase YUCCA9* (*TaYUC9*). Additionally, we investigated the NO biosynthesis gene *nitric oxide synthase 1* (*TaNOS1*) and SL biosynthesis genes *β-carotene isomerase D27* (*TaD27*), *carotenoid cleavage dioxygenase 7* (*TaMAX3*), and *cytochrome P450 711A1* (*TaMAX1*). The results revealed that both *TaTAA1* (Figure 8A) and *TaYUC9* (Figure 8B) expression levels declined drastically in XY54 and J411 at 2–8 DAT. Interestingly, *TaNOS1* expression in J411 declined significantly at 4–8 DAT compared to 0 DAT, although it was not significantly different from the LL8d control, while in XY54, it remained relatively unchanged (Figure 8C). *TaD27* expression in J411 declined to approximately the LL8d control level at 4–6 DAT, whereas in XY54, it changed only slightly (Figure 8D). We observed that *TaMAX3* expression levels declined significantly at 2–8 DAT in both varieties (Figure 8E). *TaMAX1* expression in XY54 increased significantly at 2–4 DAT, followed by a significant decrease at 8 DAT, while in J411, it decreased significantly at 8 DAT (Figure 8F). Notably, no significant difference was observed in the expression of *TaNOS1* and *TaD27* between 8 DAT and LL8d in both varieties, indicating that these genes may also be regulated by leaf age. Comprehensively, we observed that XY54 exhibited significantly higher expression of *TaMAX1* at 2–4 DAT but lower expression of *TaNOS1* and *TaD27* at 2–4 DAT compared to J411.

### 2.9. Hierarchical Clustering Analysis of Phytohormone Biosynthesis Gene Expression Patterns under Long-Term HL Stress

**Figure 9:**
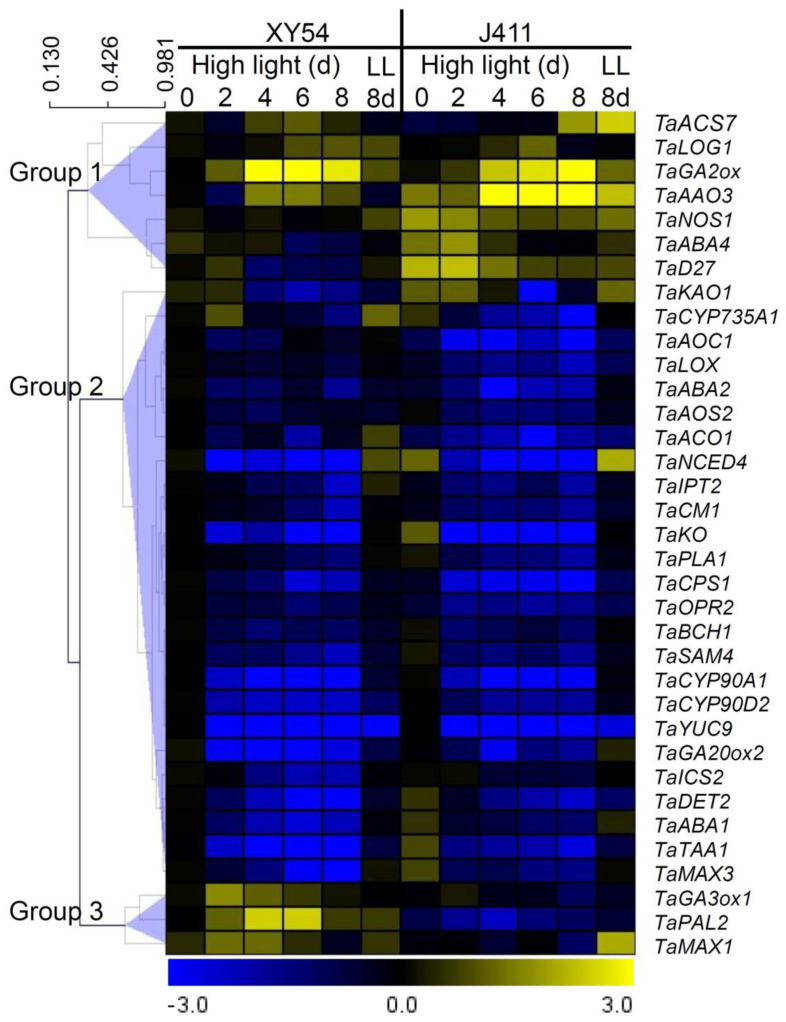
Hierarchical clustering of the expression of 35 phytohormones biosynthesis genes in XY54 and J411 subjected to HL for 2–8 d. The yellow color indicates higher expression levels of the corresponding genes, while blue indicates lower expression levels and black represents no significant change in gene expression. The clustering of genes into groups 1, 2, and 3 further highlights patterns of expression that are consistent within these groups under the experimental conditions.

To gain a comprehensive understanding of the expression patterns of phytohormone biosynthesis genes in response to HL stress, HCL on the mean expression values of the 35 investigated genes was performed (Figure 9). This analysis allowed us to categorize the genes into three distinct groups based on their expression patterns, with a distance threshold set at 0.89. The first group comprised seven genes: *TaGA2ox, TaABA4, TaAAO3, TaLOG1, TaSCS7, TaNOS1*, and *TaD27*. Notably, all genes in this group, except *TaLOG1*, exhibited higher expression levels in J411 compared to XY54. For instance, significantly higher expression of *TaNOS1* at 0–4 DAT, *TaABA4* at 2 DAT, *TaGA2ox* and *TaACS7* at 8 DAT, *TaAAO3* at 4–8 DAT, and *TaD27* at 0–4 DAT were observed in J411 relative to XY54. The second and largest group consisted of 25 genes, all of which were inhibited by HL exposure compared to the LL controls in both the XY54 and J411 varieties. This widespread downregulation suggests a common response mechanism to HL stress across multiple phytohormone biosynthesis pathways in both wheat varieties. The third group, comprising *TaGA3ox1*, *TaPAL2*, and *TaMAX1*, displayed higher expression levels in XY54 under HL conditions compared to J411. Specifically, significantly higher expression of *TaMAX1* at 2–4 DAT, *TaGA3ox1* at 2–8 DAT, and *TaPAL2* at all sampling time points was noted in XY54 relative to J411. Thus, these results highlight the significant variety-specific differences in phytohormone biosynthesis gene regulation under HL stress, potentially contributing to the differential stress responses observed between XY54 and J411 wheat varieties.

### 2.10. Correlation Analysis of Phytohormone Biosynthesis, Metabolic Changes, and Leaf Senescence under Long-Term HL Stress

To elucidate the intricate relationship between phytohormone biosynthesis and leaf senescence induced by HL stress, we conducted an extensive correlation analysis. This investigation encompassed the expression levels of 35 phytohormone biosynthesis genes, JA, GA, and ABA content and previously reported data on fructose, sucrose, starch, and chlorophyll content, as well as the expression of wheat *ADP-glucose pyrophosphorylase (TaAGP)* [27,28]. Our analysis revealed significant correlations between 26 of the 35 genes, along with JA, GA, and ABA content, and the markers of leaf senescence induced by long-term HL exposure (Table 1). Intriguingly, we observed that the expression levels of 24 genes exhibited negative correlations with fructose, sucrose, and starch content while showing positive correlations with chlorophyll content and *TaAGP* expression. In contrast, the expression levels of *TaGA2ox2* and *TaAAO3*, as well as JA, GA, and ABA content displayed positive correlations with fructose, sucrose, and starch content, but negative correlations with chlorophyll content and *TaAGP* expression. Together, these results suggest a complex interplay between phytohormone biosynthesis and metabolic changes associated with HL-induced leaf senescence. Based on these correlation patterns, it is inferred that GA, ABA, and JA may play more prominent roles than other phytohormones in regulating leaf senescence induced by long-term HL exposure.

## 3. Discussion

Plants in natural environments face diverse stresses, including HL exposure, which can induce photooxidative stress and accelerate leaf senescence, particularly in wheat flag leaves during late grain-filling stages [33,34]. This complex interplay of environmental and developmental factors poses significant challenges to studying HL-induced leaf senescence in field conditions. Understanding and improving wheat’s tolerance to HL stress is crucial for enhancing radiation use efficiency (RUE) and genetic yield potential, thereby addressing the growing global demand for food security. Plant hormones play a pivotal role in coordinating plant responses to environmental stimuli and developmental processes, serving as essential regulators of stress resilience and crop productivity [35]. The fundamental signaling pathways governing phytohormone effects have been extensively elucidated through genetic screens and targeted research approaches, with recent interactome studies further expanding our understanding of selective pathways [36]. Here, in this study, photosynthetic activity, and the roles of phytohormones in wheat HL tolerance regulation were systematically investigated. The transcriptional responses of 35 genes from 10 phytohormone biosynthesis pathways to long-term HL exposure were studied, revealing their involvement in HL-induced leaf senescence. Hierarchical clustering analysis grouped these genes into three distinct clusters based on their expression patterns. Notably, 25 genes were significantly repressed by HL, while others showed variety-specific responses, with some highly expressed in the HL-sensitive J411 and others in the HL-tolerant XY54.

First, sustained strong HL stress causes photooxidation-induced senescence. In this study, when wheat plants were transferred from LL to HL for 6–8 days, a rapid decline in chlorophyll a + b and chlorophyll a was observed, indicating significant chlorophyll degradation and leaf aging. Initially, carotenoid (Car) content increased, suggesting an activation of the protective mechanisms against strong light stress; however, Car content later decreased at 6 and 8 days. The reduction in chlorophyll content under strong light reflects decreased photosynthetic pigment levels and impaired photosynthesis, while the Car increase indicates a stress response. Similarly, HL exposure for more than 2 days led to significant reductions in Fv/Fm, PI, ETo/CS, and RC/CSm, while DIo/CS increased, reflecting decreased photochemical efficiency, electron transport, and reaction center density, with increased heat dissipation. Compared to plants grown under LL, those under HL had markedly lower Fv/Fm values, demonstrating impaired photosynthesis and severe photooxidative stress. These results indicate that prolonged strong HL exacerbates leaf photooxidation and aging; however, enhanced heat dissipation does provide a protective response.

Bioactive GA is an essential element that plays an active role in delaying leaf senescence in plants subjected to stressful environments. Initially, GA was predominantly viewed as a negative regulator of leaf senescence [37,38]. However, recent studies have challenged this perspective, suggesting that GA may positively influence leaf senescence in Arabidopsis [39,40,41]. The investigation into GA biosynthesis genes under HL stress revealed a complex regulatory pattern. Genes such as *TaCPS1*, *TaKO*, *TaKAO1*, and *TaGA20ox2* were inhibited by long-term HL exposure in both XY54 and J411 wheat varieties, while the GA catabolizing gene *TaGA2ox* was induced (Figure 3). These findings align with previous reports and suggest a potential decline in biologically active GA under HL conditions for both varieties [42]. Interestingly, variety-specific response in *TaGA3ox1* expression was observed, with XY54 showing significantly higher levels at 2–6 DAT compared to LL controls, while J411 exhibited minimal changes under HL. This observation is particularly noteworthy, as *GA3ox* catalyzes the rate-limiting step in GA biosynthesis, converting GA9 to GA4, the primary bioactive GA in Arabidopsis [43]. This differential expression pattern implies that GA may play a crucial role in regulating HL tolerance in XY54, highlighting the complex and variety-specific nature of GA-mediated responses to HL stress in wheat. Next, we measured bioactive GA content in XY54 and J411 varieties under both normal and stressed conditions, finding a significant reduction after prolonged exposure to HL. This decrease in GA levels may result from several potential mechanisms, including, as follows: a shortage of upstream substrates in the GA biosynthesis pathway; post-transcriptional modifications affecting GA20ox and GA3ox genes; and suppressed expression of GA biosynthesis-related genes upstream of GA20ox [44].

The regulation of ABA biosynthesis is crucial for plant stress responses and leaf senescence, with *9′-cis-epoxy carotenoid dioxygenase* (*NCED*) playing a key role. For instance, in rice, studies have shown that overexpression of *OsNCED5* increases ABA levels and stress tolerance while accelerating leaf senescence [45]. This study revealed a complex pattern of ABA pathway gene regulation under HL stress in wheat. While *TaNCED4*, *TaBCH1, TaABA1*, *TaABA4,* and *TaABA2* were repressed by HL in both XY54 and J411 varieties, *TaAAO3* was notably induced, particularly in the HL-sensitive J411 (Figure 5). This aligns with previous findings in tomatoes, where *ABA*1 overexpression enhanced sensitivity to HL and chilling stress [7,8]. Furthermore, AAO catalyzes the final step in ABA biosynthesis, simultaneously elevating ABA and reactive oxygen species levels [46]. In Arabidopsis, increased *AAO3* activity coincided with higher ABA levels and premature senescence [47,48]. Therefore, the higher expression of *TaABA1* and *TaAAO3* in J411 under HL conditions suggests a potential mechanism for its increased HL sensitivity, possibly leading to consistently higher ABA and ROS levels and accelerated leaf senescence compared to XY54.

High light activates ABA signaling in plants, leading us to investigate whether increased ABA accumulation was responsible for this signaling. To address this, we measured endogenous ABA levels in both varieties grown under HL stress. The results showed that ABA content significantly increased in both varieties under HL stress, supporting the notion that plants enhance ABA accumulation in response to HL, which in turn activates ABA signaling (Figure S1A). Overexpressing sweet potato *IbABF4* in Arabidopsis and sweet potato enhanced tolerance to abiotic stress, resulting in lower ROS levels, increased ABA content, and higher expression of stress-associated LEA genes [49]. Notably, the higher ABA content in the XY54 variety under HL conditions may trigger more ABA-responsive genes related to ROS scavenging, potentially offering greater protection against HL stress.

Furthermore, the regulation of CK levels plays a crucial role in leaf senescence, with enhanced CK levels through the overexpression of CK biosynthesis genes, such as *IPT*, that are known to retard leaf senescence [50,51]. In this study, the expression levels of two CK biosynthesis genes, *TaIPT2* and *TaCYP735A1*, decreased consistently in both XY54 and J411 varieties when subjected to HL for 4–8 days (Figure 6A,B), aligning with the previously observed weak expression of CK biosynthesis genes during senescence [42]. However, *TaLOG1* expression increased at 6 DAT in J411 and 6–8 DAT in XY54 (Figure 6C). *LONELY GUY* (*LOG*), encoding riboside 5′-monophosphate phosphoribohydrolase, catalyzes the final step of bioactive CK synthesis; its overexpression in Arabidopsis has been shown to promote cell division, reduce apical dominance, and retard leaf senescence [52]. The induction of *TaLOG* may counteract the repression of *TaIPT2* and *TaCYP375A1*, potentially maintaining CK levels under HL conditions. Notably, *TaCYP375A1* expression in XY54 increased significantly at 2 DAT before consistently declining with prolonged HL exposure, whereas J411 exhibited a dramatic and consistent decline from 2 to 8 DAT. The higher expression of *TaIPT2* and *TaCYP375A1* at 2 DAT and *TaLOG1* at 8 DAT in XY54 may contribute to elevated CK levels in this variety compared to J411 in response to HL.

Similarly, the ET biosynthesis genes exhibited inconsistent responses to HL, potentially due to variations among gene family members. Van der Graaff et al. (2006) reported increased transcripts for about 25% of ET synthesis and signaling genes during senescence and observed diverse responses. For instance, *TaSAM4* was repressed by HL in both XY54 and J411 (Figure 6D). The induction of *TaACS7* in J411 at 8 DAT aligned with the upregulation of *AtACS2*, *AtACS5*, *AtACS6,* and *AtACS7* during natural senescence in Arabidopsis [42,53]. However, *TaACS7* expression in J411 at 8 DAT, while increased compared to 0 DAT, was significantly lower than the LL8d control, suggesting that leaf age rather than HL primarily influenced its expression. Conversely, *TaACO1* expression declined at 2–8 DAT in both varieties compared to the LL8d control, in contrast with previous findings [42]. These results indicate that *TaACO1* may respond differently to natural senescence in Arabidopsis versus HL-induced senescence in wheat.

Salicylic acid biosynthesis, known for its role in plant immune defense, involves either the isochorismate or phenylpropanoid pathway. In Arabidopsis, *ICS1*, encoding isochorismate synthase, participates in both SA biosynthesis and phylloquinone synthesis, influencing chloroplast ultrastructure, plastoquinone pool redox status, photosystem organization, and light acclimation [54]. Here, *TaICS2* and *TaCM1*, involved in the isochorismate pathway, were consistently suppressed by HL in both varieties. However, *TaPAL2* expression in XY54 increased substantially at 2–6 DAT while remaining stable in J411, suggesting that the phenylpropanoid pathway may contribute more significantly to SA biosynthesis in wheat under HL conditions. The higher expression of *TaPAL2* in XY54 under HL indicates that the phenylpropanoid pathway may play a more prominent role in SA biosynthesis in this HL-tolerant variety, in contrast with the HL-sensitive variety J411. In addition, BR has been found to positively regulate leaf senescence in Arabidopsis [22] and *Carica papaya* [23], although BR analogs advanced leaf senescence in wheat [24]. Furthermore, BR biosynthesis genes *TaCYP90A1*, *TaDET2*, and *TaCYP90D2* were consistently repressed by HL in both XY54 and J411, indicating a negative relationship between BR synthesis gene expression and HL-induced leaf senescence. Likewise, IAA biosynthesis genes *TaTAA1* and *TaYUC9* were markedly inhibited by HL in both varieties (Figure 7A,B). The lack of significant differences between XY54 and J411 in BR and IAA biosynthesis gene expression suggests minimal roles for these hormones in regulating HL tolerance in XY54.

Furthermore, NO biosynthesis, represented by *TaNOS1* expression, was inhibited in J411 at 4–8 DAT but remained relatively stable in XY54 under HL conditions (Figure 8C). Nitric oxide has been shown to play counteractive roles in regulating dehydration-induced senescence in rice [55], ABA-induced senescence in Arabidopsis [56], and dark-induced senescence in Arabidopsis [57]. The inhibition of *TaNOS1* in J411 at 4–8 DAT may lead to decreased NO levels and earlier senescence when subjected to long-term HL. To this end, SL has been found to promote leaf senescence [58,59,60]. The expression of *TaMAX3*, an SL biosynthesis gene, declined consistently in both XY54 and J411 at 2–8 DAT. However, *TaD27* and *TaMAX1* responded differently to HL in the two varieties. The expression of *TaD27* in J411 declined significantly at 6–8 DAT but remained similar to the LL8d control, while it changed slightly in XY54, suggesting that *TaD27* expression was determined more by leaf age than HL.

Next, all five investigated JA biosynthesis genes (*TaPLA1*, *TaLOX*, *TaAOS2*, *TaAOC1*, and *TaOPR2)* were repressed in both XY54 and J411 when exposed to HL conditions (Figure 4). Notably, *TaAOS2* expression levels in XY54 at 6–8 DAT were similar to the LL8d control, while in J411, they declined significantly at 2–8 DAT. Conversely, *TaAOC* and *TaOPR2* expression in XY54 declined significantly at 2–8 DAT, while in J411, they changed only slightly under HL compared to the LL8d control. These observations suggest that *TaAOS2* expression in XY54, as well as *TaAOC1* and *TaOPR2* expression in J411, may also be regulated by leaf age. Collectively, the higher expression of *TaPLA1* at 2–4 DAT, *TaLOX* at 2–8 DAT, *TaAOS2* at 6–8 DAT, *TaAOC1* at 2–4 and 8 DAT, and *TaOPR2* at 2–4 and 8 DAT in XY54 demonstrated that JA biosynthesis was more pronounced in XY54 compared to J411 under HL conditions. Additionally, JA content increased drastically in both varieties when exposed to HL. A recent study has shown that JA positively regulates the response of Arabidopsis to HL and heat stress through the activation of JA-responsive genes such as those involved in ROS scavenging [17]. The higher JA content in XY54 under HL conditions may activate more JA-responsive genes involved in ROS scavenging, potentially protecting XY54 against HL stress, despite JA being generally considered to promote leaf senescence [61]. Further research is needed to elucidate the precise roles of JA in regulating HL tolerance in wheat.

Correlation analysis revealed that the expression levels of 24 out of 35 investigated phytohormone biosynthesis genes were negatively correlated with fructose, sucrose, and starch content, but were positively correlated with chlorophyll content and *TaAGP* expression. These genes, which were repressed by HL, demonstrated a negative relationship with HL-induced leaf senescence. In contrast, the expression levels of *TaGA2ox2* and *TaAAO3*, as well as JA content, exhibited positive correlations with fructose, sucrose, and starch content, while showing negative correlations with chlorophyll content and *TaAGP* expression. Consequently, the induction of *TaGA2ox2* and *TaAAO3* expression, along with elevated JA content, displayed a positive relationship with HL-induced leaf senescence. Given that *GA2ox* degrades GA while *AAO* synthesizes ABA, the decline in GA levels, coupled with the enhancement of ABA and JA levels, were hypothesized to be positively associated with sugar accumulation during long-term HL-induced leaf senescence in wheat. Thus, the significant interactions suggest that XY54 and J411 respond differently to prolonged HL stress, with the variety-specific regulation of phytohormone biosynthesis genes playing a central role in determining each variety’s tolerance to HL-induced leaf senescence.

## 4. Materials and Methods

### 4.1. Plant Material and Growth Conditions

Two Chinese winter wheat (*Triticum aestivum* L.) cultivars, Xiaoyan 54 (XY54) and Jing 411 (J411) were utilized in this study, with plant cultivation and HL treatment conducted as previously described [27,28,62]. One week after germination, the plants were cultivated in a nutrient medium and grown in a growth chamber (HP1000GS, Wuhan Ruihua Instrument & Equipment Co., China). At the three-leaf stage, plants grown under low light (LL) conditions of 300 μmol m^−2^ s^−1^ photosynthetic photon flux density (PPFD) were subjected to HL conditions of 800 μmol m^−2^ s^−1^ PPFD for 2–8 days. The first leaves were collected for analysis at 0, 2, 4, 6, and 8 days after transfer (DAT) to HL conditions; the first leaf samples from plants maintained under LL conditions for 8 days (LL8d) served as the LL control. For each sampling time point and cultivar, the first leaf from a single plant was collected. At least three independent biological repeats were carried out and eight leaves were randomly collected for each cultivar.

### 4.2. Determination of Chlorophyll and Carotenoid Contents

The total chlorophyll content (Chl a + b) and carotenoids (Car) in wheat leaves were determined using Arnon’s (1949) method. Approximately 0.05 g of a leaf sample was placed in a 1.5 mL centrifuge tube with 1 mL of 80% acetone. The tube was sealed to prevent acetone evaporation and stored in the dark at room temperature for 3 days, allowing complete extraction until the leaf tissue turned white. After extraction, the sample was centrifuged at 2000 rpm for 5 min. The supernatant was then analyzed using a full-wavelength microplate reader (Spectra Max 190, Meigu Molecular Instruments (Shanghai) Co., Ltd.), with optical density (OD) measurements taken at wavelengths of 470 nm, 645 nm, 646 nm, and 663 nm. The experiments were performed in triplicate, and the levels of chlorophyll a, chlorophyll b, total chlorophyll (Chl a + b), and total carotenoids were measured using the following formulas:

Chlorophyll a (Chl a):

Chl a (mg/L) = 12.7 × A663 − 2.69 × A645

Chlorophyll b (Chl b):

Chl b (mg/L) = 22.9 × A645 − 4.68 × A663

Total Chlorophyll (Chl a + b):

Chl a + b (mg/L) = 20.2 × A645 + 8.02 × A663

Carotenoids:

Car (mg/L) = (1000 × A470 − 1.82 × Chl a − 85.02 × Chl/198)

### 4.3. Chlorophyll Fluorescence Measurement

Chlorophyll fluorescence measurements were carried out with a Pulse-Amplitude-Modulation (PAM) fluorometer (PAM-2100, Walz, Effeltrich, Germany). The entire plant seedlings were dark-treated for 30 min. Then, the minimal fluorescence yield of the dark-adapted state (Fo) and the maximal fluorescence yield of the dark-adapted state (Fm) of rosette leaves were measured. The maximal quantum yield of PSII photochemistry (Fv/Fm) was calculated as Fv/Fm = (Fm − Fo)/Fm [63]. The intensity of continuous actinic illumination was adjusted to 200 μmol m^−2^ s^−1^ for 5 min. Then, a saturating pulse was applied to measure the maximal fluorescence yield of the light-adapted state (Fm′) and the steady-state fluorescence (Fs). Far-red light was subsequently used to record the minimal fluorescence yield of the light-adapted state (Fo′). The actual quantum yield of PSII (Y(II)) was calculated as Y(II) = (Fm′ − Fs)/Fm′ [64]. The photochemical quenching coefficient (qP) was calculated as qP = (Fm′ − Fs)/(Fm′ − Fo′) [65]. Four replicates were performed.

### 4.4. RNA Isolation and First-Strand cDNA Synthesis

Total RNA was extracted with TRIzol reagent (Thermo Fisher Scientific, USA) following the manufacturer’s instructions. After leaf samples were grounded to fine powder in liquid nitrogen, about 0.01 g leaf powder was used to extract total RNA with 1 mL of TRIzol (Life Technologies, Carlsbad, CA, USA) reagent. Total RNA was quantified with a spectrophotometer Nanodrop 2000 (Thermo Scientific, Waltham, MA, USA). First-strand cDNA was synthesized with an Evo M-MLV Reverse Transcriptase Kit with gDNA Clean (Accurate Biotechnology, Changsha, China) following the manufacturer’s instructions. First, 10 µL of the reaction solution, including 2 μg total RNA, 1 μL gDNA Clean Reagent, 5 μL 5× gDNA Clean Reagent Buffer, and an appropriate amount of RNase-free water, was incubated at 42 °C for 2 min. Second, 10 μL of the solution, consisting of 1 μL Evo M-MLV reverse transcriptase mix, 1 μL of 50 μM Oligo(dT)18 primer, 1 μL of 100 μM 6-mers random primer, 4 μL 5× reverse transcriptase reaction buffer, and 3 μL RNase free water, was mixed with the first step 10 μL solution, followed by incubation at 37 °C for 15 min. Then, the reaction was terminated by incubation at 85 °C for 5 s. Finally, 60 μL RNase free water was added as a PCR template.

### 4.5. Quantification of Gene Expression

Quantitative polymerase chain reaction (qPCR) was performed by using StepOnePlusTM Real-Time PCR Systems (Thermo Fisher Scientific, USA) to assay the relative expression of genes. It was carried out with 10 μL of a qPCR reaction solution consisting of 2 μL cDNA, 5 μL of 2× PowerUp SYBR Green Master Mix (Thermo Fisher Scientific, USA), and 0.2 μL of the gene-specific primers following a three-step PCR program. The gene-specific primers were designed according to Chinese Spring reference genes by using Primer Express (version 3.01). The oligonucleotide primers were synthesized by Sangon Biotech Co., Ltd. (Shanghai, China). The gene-specific primer sequences are shown in Table S1. The TaActin was used as an internal reference gene. Four replicates of each sample and gene expression were quantified following the CT relative quantification method [66]. The relative expression levels for each gene were computed by using the expression levels of XY54 before HL treatment (HL0d) as a control. The expression patterns of some of the investigated genes were confirmed by using *glyceraldehyde-3-P dehydrogenase* (*GAPDH*) as the second internal reference gene.

### 4.6. Jasmine Acid, Gibberellins, and Abscisic Acid, Quantification

Fresh plant material (50 mg) was immediately frozen in liquid nitrogen and stored at −80 °C until used. JA, GA, and ABA were extracted and purified as described by Fu et al. (2012) with some modifications. About 50 g of a leaf sample was homogenized and extracted for 24 h in methanol and isotopically labeled. After purification was conducted with an Oasis Max solid phase extract cartridge, LC-MS/MS (Agilent 6495 tandem triple quadrupole mass spectrometer Santa Clara, CA, USA) the analysis was carried out on a UPLC system (Waters, Shanghai, China) coupled to the 5500 Q-Trap system (AB SCIEX). The sample was injected into a BEH C18 column (1.7 mm, 2.1 × 150 mm; Waters) with mobile-phase 0.05% acetic acid (A) and 0.05% acetic acid in acetonitrile (B). The hormone contents, including ABA, GA, and JA was determined using the HPLC-UV external standard method, which was completed by Suzhou Gris Biotechnology Co., Ltd. (Suzhou, China). Three biological replicates were performed for each treatment.

### 4.7. Hierarchical Clustering Analysis

The mean values of the expression levels were log-transformed before hierarchical clustering (HCL) analysis was carried out. It was performed with the cosine correlation distance metric and average linkage clustering method by using the software Multiple Array Viewer (version 4.9) [67].

### 4.8. Data Analysis

The experiment was designed as a completely randomized design (CRD) with three biological replicates for each treatment. Data are presented as mean ± standard error (SE). A factorial design was employed. For statistical analysis, a one-way analysis of variance (ANOVA) was performed to assess the significance of differences among treatment means. Tukey’s post hoc test was applied for pairwise comparisons at *p* ≤ 0.05. Additionally, correlation analyses were conducted. All statistical analyses were performed using IBM SPSS statistical software (version 19.0), with data visualization and plotting carried out using SigmaPlot (version 10.0).

## 5. Conclusions

This study highlights the crucial role of phytohormones in wheat’s response to HL stress, mainly focusing on their involvement in leaf senescence. The results indicate that prolonged HL exposure leads to a significant decline in chlorophyll content and photosynthetic performance, with sensitive varieties, such as J411, showing more pronounced effects than tolerant varieties such as XY54. The differential expression of GA, ABA, and JA biosynthesis genes was found to be vital in mediating these responses, with XY54 exhibiting higher ABA and JA levels, which may contribute to its enhanced tolerance. Moreover, the study revealed a complex regulatory pattern of GA biosynthesis genes, suggesting that the interplay between reduced GA levels and increased ABA and JA levels might initiate sugar accumulation and leaf senescence under HL stress. Together, these results deepen our understanding of the hormonal regulation of HL stress tolerance in wheat and could guide future efforts to improve crop resilience.

## Figures and Tables

**Table 1:** Correlation coefficients between the expression levels of phytohormones biosynthesis genes and the content of fructose, sucrose, starch, and chlorophyll and the expression levels of *TaAGP* encoding ADP-glucose pyrophosphorylase responding to long-term HL.

Phytohormones	Genes	Fructose	Sucrose	Starch	Chlorophyll	*TaAGP*
Gibberellins	*TaCPS1*	−0.821 **	−0.817 **	−0.786 **	0.678 **	0.727 **
	*TaKO*	−0.832 **	−0.789 **	−0.775 **	0.721 **	0.853 **
	*TaKAO1*	−0.461 **	−0.450 **	−0.510 **	0.673 **	0.591 **
	*TaGA20ox2*	−0.764 **	−0.679 **	−0.696 **	0.655 **	0.823 **
	*TaGA2ox*	0.679 **	0.657 **	0.758 **	−0.766 **	−0.721 **
GA content	*-*	0.859 **	0.759 **	0.794 **	−0.691 **	−0.822 **
Cytokinins	*TaIPT2*	−0.795 **	−0.782 **	−0.779 **	0.611 **	0.684 **
	*TaCYP735A1*	−0.664 **	−0.698 **	−0.699 **	0.646 **	0.584 **
Ethylene	*TaSAM4*	−0.841 **	−0.801 **	−0.818 **	0.764 **	0.894 **
Salicylic acid	*TaICS2*	−0.599 **	−0.544 **	−0.636 **	0.691 **	0.719 **
	*TaCM1*	−0.788 **	−0.834 **	−0.816 **	0.739 **	0.675 **
Abscisic acid	*TaBCH1*	−0.788 **	−0.720 **	−0.719 **	0.625 **	0.829 **
	*TaABA1*	−0.837 **	−0.759 **	−0.789 **	0.785 **	0.935 **
	*TaNCED4*	−0.794 **	−0.734 **	−0.718 **	0.690 **	0.740 **
	*TaABA2*	−0.750 **	−0.778 **	−0.698 **	0.552 **	0.616 **
	*TaAAO3*	0.469 **	0.609 **	0.538 **	−0.497 **	−0.288 *
ABA content	*-*	0.816 **	0.788 **	0.688 **	−0.767 **	−0.798 **
Brassinosteroids	*TaCYP90A1*	−0.936 **	−0.873 **	−0.867 **	0.784 **	0.921 **
	*TaDET2*	−0.703 **	−0.669 **	−0.723 **	0.746 **	0.860 **
	*TaCYP90D2*	−0.794 **	−0.731 **	−0.753 **	0.699 **	0.875 **
Indole-3-acetic acid	*TaTAA1*	−0.706 **	−0.647 **	−0.673 **	0.656 **	0.863 **
	*TaYUC9*	−0.660 **	−0.623 **	−0.628 **	0.565 **	0.744 **
Strigolactones	*TaMAX3*	−0.820 **	−0.778 **	−0.788 **	0.782 **	0.904 **
Jasmonic acid (JA)	*TaLPLA1*	−0.878 **	−0.895 **	−0.881 **	0.822 **	0.827 **
	*TaLOX*	−0.579 **	−0.650 **	−0.603 **	0.422 **	0.430 **
	*TaAOS2*	−0.701 **	−0.721 **	−0.639 **	0.523 **	0.655 **
	*TaAOC1*	−0.559 **	−0.567 **	−0.498 **	0.305 *	0.327 *
	*TaOPR2*	−0.673 **	−0.688 **	−0.635 **	0.463 **	0.527 **
JA content	-	0.759 **	0.694 **	0.777 **	−0.747 **	−0.806 **

Note: * and ** denote the significance at *p* < 0.05 and *p* < 0.01, respectively. The data for the content of fructose, sucrose, starch, and chlorophyll and the expression of *TaAGP* in XY54 and J411 subjected to HL for 2–8 d are from [27,28].

## Data Availability

Data are contained within the article and Supplementary Materials.

## Supplementary Materials

The following supporting information can be downloaded at: https://www.mdpi.com/article/10.3390/plants13182628/s1, Figure S1: GA and ABA content under HL stress. GA and ABA content in XY54 and J411 responding to leaf senescence induced by HL. The data are represented as mean ± SE. The different letters indicate a significant difference at *p* < 0.05; Table S1: qPCR primer sequences used in this study (the primer sequences are shown as 5′→3′).
