# Supplementary material for: Comparative Analysis of Phytohormone Biosynthesis Genes Responses to Long-Term High Light in Tolerant and Sensitive Wheat Cultivars
Source: Plants (Basel). 2024 Sep 20;13(18):2628. doi: 10.3390/plants13182628 (PMC11435395; doi:10.3390/plants13182628)
Supplement: Supplementary file 1 [file plants-13-02628-s001.zip › plants-3184508-supplementary.pdf]

Supplementary information

Table S1. qPCR primer sequences used in this study (the primer sequences are shown as 5'→3').

Phytohormones	Genes	qPCR primers [a]	Chinese Spring reference genes [b]	Annotation
Gibberellins	*TaCPS1*	TTCTAGAACGGACCGAGGAA	TraesCS7B02G476400	*ent*-copalyl diphosphate synthase 1
		TGGTTGTCGAGAATCCACTG	TraesCS7D02G539200	
	TaKO	AGAAGTTTTCAAGGACGAGCTGTT	TraesCS7A02G362300	*ent*-kaurene oxidase
		CCCACATCCTCGCCTAAACTC	TraesCS7B02G265800	
			TraesCS7D02G360700	
	TaKAO1	ACGAGGAGGTGGTGGACAAC	TraesCS5D02G004900	*ent*-kaurenoic acid oxidase 1
		GCTGGCGGTAGACTCGTATCC		
	TaGA20ox2	ATTCTGCTCTGCCACGAAAC	TraesCS3D02G401400	Gibberellin 20 oxidase 2
		CCAAAAATGGAAGGTGGTGT		
	TaGA3ox1	CAGCTTCAACCAGCTCAACGA	TraesCS4A02G109200	GA3-oxidase 1
		AGCTCCCGGAACAAACCCT	TraesCS4B02G003300	
			TraesCS4D02G002800	
	TaGA2ox	CGTGTGACGTGTGTGTTCATAGTT	TraesCS3A02G294000	GA2-oxidase
		CGTTCGTCCGGCATTCC	TraesCS3B02G328700	
			TraesCS3D02G293800	
Cytokinins	*TaIPT2*	GAATGGGAACAGCACAAGCA	TraesCS2A02G265700	tRNA isopentenyl transferase 2
		TCCGCTTCAAACGCTGTGT		
	TaCYP735A1	CTCCTCACCTGGGCCATC	TraesCS7A02G324300	Cytokinin hydroxylase
		AGCGTCTCGTTGATCACCAT	TraesCS7B02G224900	
	TaLOG1	TGAAGCTTCTCCCCCAGATA	TraesCS3A02G251500	Cytokinin riboside 5'-monophosphate phosphoribohyd
		GCCTGGCTTGTTTCCTCTC	TraesCS3B02G281000	

			TraesCS3D02G251900	rolase 1
Ethylene	*TaSAM4*	GTGGTGCATATGTTGCAAGG	TraesCS6A02G169300	S-adenosylmethionine synthase 4
		TCGAGGATCTCCTTGTCAGG		
	TaACS7	GGTGTGCTTCGCCAA	TraesCS3A02G106600	1-aminocyclopropane-1-carboxylate synthase 7
		GCCTTGTTCCACCTGTCCAT	TraesCS3B02G125200	
			TraesCS3D02G108700	
	TaACO1	CCACGCCATAGTCGTCAACA	TraesCS6A02G325500	1-aminocyclopropane-1-carboxylate oxidase 1
		GCGGTGCATCACGCTCTT	TraesCS6D02G305100	
Salicylic acid	*TaICS2*	CAAACCGCCATCGTAAATCT	TraesCS5A02G193800	Isochorismate synthase 2
		GGGTGGCTGTATGCAGAACT	TraesCS5B02G189100	
			TraesCS5D02G196200	
	TaCM1	GGGAGATACAAGAGCCCAGAT	TraesCS3A02G301800	Chorismate mutase
		GCAGGTCGTGTCGCAAAG	TraesCS3D02G300200	
	TaPAL2	TGAACCAGGGCAAGCACAT	TraesCS1A02G037700	Phenylalanine ammonia-lyase 2
		AGGGCTCACCATTCCACTCTTT	TraesCS1B02G048100	
			TraesCS1D02G039400	
Abscisic acid	*TaBCH1*	GCTGCCGCTCACAAGATA	TraesCS2D02G393900	Beta-carotene hydroxylase B1
		GCTCCTGCTCCAGTTCGTC		
	TaABA1	GGCGAAACGGATGCATCTT	TraesCS2A02G317000	Zeaxanthin epoxidase
		AGGAGGCACAAAGTCTGCAATT	TraesCS2B02G335400	
			TraesCS2D02G314900	
	TaABA4	GTTCGCGAGCGAGATGAC	TraesCS3A02G043800	Protein ABA DEFICIENT 4
		ATCCCAATGGGGCAGAAG		

	TaNCED4	GGGGAGCCGTTTTTCGTC	TraesCS6A02G271600	9-*cis*-epoxycarotenoid dioxygenase
		CGTCCATCACCACGAACC	TraesCS6B02G298800	
	TaABA2	GCTGCATTTGTTACTTGGTATCTGA	TraesCS5A02G465100	Xanthoxin dehydrogenase
		CACCCACCTTATTGACATGCTAAA	TraesCS5B02G476900	
			TraesCS5D02G477700	
	TaAAO3	CTTTCGTCAAAAGCCTCTGG	TraesCS7B02G417900	Aldehyde oxidase
		TATCCACGATGTCCAAACCA	TraesCS7D02G498100	
Brassinosteroids	*TaCYP90A1*	AGCGTTGAGGGAGGTGAT	TraesCS5A02G131400	Cytochrome P450 90A1
		AGCAGGGACAGGCAGAAG	TraesCS5B02G133400	
			TraesCS5D02G139900	
	TaDET2	CTACTAGCCTCTGCAACTTCT	TraesCS3D02G359100	Steroid 5-alpha-reductase DET2
		AATCAGCCAATCCTCAATC		
	TaCYP90D2	CGCCGTCAAGTTCCTCAG	TraesCS3A02G103800	Cytochrome P450 90D2
		CAGCGTCTCCGTTATCACAT	TraesCS3B02G121200	
			TraesCS3D02G106100	
Jasmonic acid	*TaPLA1*	CAAAGAAGGGTCTGCGCATT	TraesCS2A02G220900	Phospholipase A1
		GACAGTTGCAAGGCCC	TraesCS2B02G246500	
			TraesCS2D02G226600	
	TaLOX	CGGAGACAGACCCAAGAAAG	TraesCS2A02G315100	Lipoxygenase
		CACGAGTTGCAGGTGAAGTG		
	TaAOS2	ACCAAGGCCGCCAAGTG	TraesCS4A02G061800	Allene oxide synthase 2
		CAAAGGGAGGCCGAACGT	TraesCS4B02G237500	
			TraesCS4D02G238700	
	TaAOC1	GAGGCCATCTACAGCATCT	TraesCS6A02G334800	Allene oxide cyclase 1
		GGTGACGGCGAGGTAGG	TraesCS6B02G3	

		A	65200	
			TraesCS6D02G314300	
	TaOPR2	CGCCGGCCTCAACAAGTA	TraesCS7A02G412400	12-oxophytodienoate reductase 2
		ACGGGTAGTCGGTGTAG	TraesCS7B02G311600	
			TraesCS7D02G405500	
Indole-3-acetic acid	*TaTAA1*	CCATGACCTCGCCTACTACTG	TraesCS3A02G093000	L-tryptophan--pyruvate aminotransferase 1
		TGTTGAGCTCCACGAACTTG	TraesCS3B02G108200	
			TraesCS3D02G093300	
	TaYUC9	AGACGACTAACCAAATCCCCT	TraesCS2A02G011500	Indole-3-pyruvate monooxygenase YUCCA9
		GAGGGCTCCGCTTGTAGTAT	TraesCS2D02G012100	
Nitric oxide	*TaNOS1*	GGCAAGCAGTTTGTTTCG	TraesCS6A02G013600	Nitric oxide synthase 1
		GACGCTAAGGACATTGAGTT	TraesCS6D02G017000	
Strigolactones	*TaD27*	TGATGATGCCACCTTCAAAA	TraesCS7A02G418900	Beta-carotene isomerase D27
		AACCTGCATTTGGGGATGTA	TraesCS7B02G319100	
	TaMAX3	GACCACGCCGGTCTACCT	TraesCS2A02G414600	Carotenoid cleavage dioxygenase 7
		GGAACCAGTCGTAGGAGCAG	TraesCS2B02G433800	
			TraesCS2D02G411900	
	TaMAX1	GTCCTCGCAAAGAAGTACGG	TraesCS4A02G412100	Cytochrome P450 711A1
		ACGGCTGGTAGATGGAGATG	TraesCS4B02G312300	
			TraesCS4D02G309900	

[a], the upper denotes the forward primer sequence, the lower indicates the reverse primer sequences.

[b], the targeted Chinese Spring reference genes for designing qPCR primers.

Supplementary figure S1

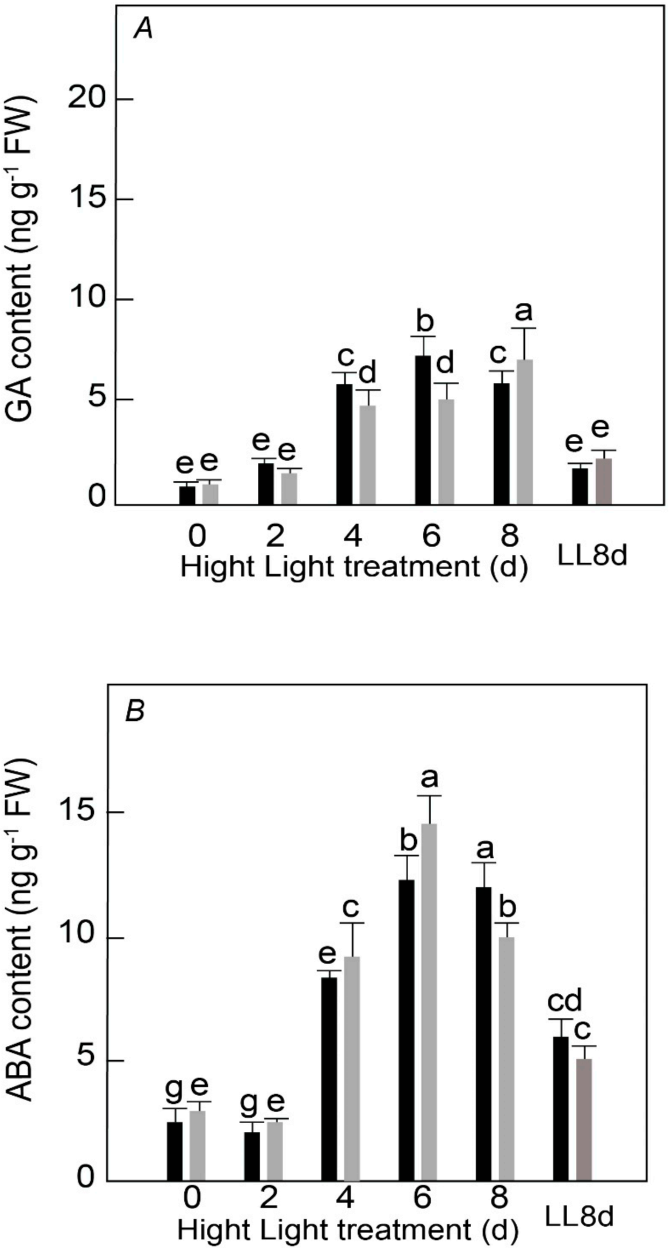

Figure S1. GA and ABA content under HL stress: GA and ABA content in XY54 and J411 responding to leaf senescence induced by HL. The data are represented as mean ± SE. The different letters indicate a significant difference at $P < 0.05$.
